# Supplementary material for: Transformations, trajectories, and similarities of national production structures: A comparative fingerprinting approach
Source: PLoS One. 2023 Dec 19;18(12):e0295568. doi: 10.1371/journal.pone.0295568 (PMC10729992; doi:10.1371/journal.pone.0295568)
Supplement: S1 Appendix — Tables, metadata and two additional fingerprinting analyses pertaining to the article. (PDF) [file pone.0295568.s001.pdf]

APPENDIXES: TRANSFORMATIONS, TRAJECTORIES, AND SIMILARITIES OF NATIONAL PRODUCTION STRUCTURES

Contents

Appendix A: Tables and metadata 2
- A.1 ISO3-to-country table 2
- A.2 Economic sectors 3
- A.3 Data coverage in WIOD13 4

Appendix B: Cluster memberships 5
- B.1 Average-link (UPMGA) 5-cluster partitions 5
- B.2 Average-link (UPMGA) 11-cluster partitions 6
- B.3 Average cluster distances 7

Appendix D: Additional analyses 8
- D.1 Dominant eigenvalues of WIOD13 fingerprints 8
- D.2 Analyzing structural trajectories using multidimensional scaling: the case of the European Union 1995-2011 11

Appendix A: Tables and metadata

A.1 ISO3-to-country table

ISO3	Country name	ISO3	Country name
AUS	Australia	IRL	Ireland
AUT	Austria	ITA	Italy
BEL	Belgium	JPN	Japan
BGR	Bulgaria	KOR	South Korea
BRA	Brazil	LTU	Lithuania
CAN	Canada	LUX	Luxembourg
CHN	China	LVA	Latvia
CYP	Cyprus	MEX	Mexico
CZE	Czech Republic	MLT	Malta
DEU	Germany	NLD	The Netherlands
DNK	Denmark	POL	Poland
ESP	Spain	PRT	Portugal
EST	Estonia	ROU	Romania
FIN	Finland	RUS	Russia
FRA	France	SVK	Slovakia
GBR	Great Britain	SVN	Slovenia
GRC	Greece	SWE	Sweden
HUN	Hungary	TUR	Turkey
IDN	Indonesia	TWN	Taiwan
IND	India	USA	United States of America

A.2 Economic sectors

Sectorial label	ISIC3's	WIOD sector description
Agriculture	A, B	Agriculture, Hunting, Forestry and Fishing
Mining	C	Mining and Quarrying
Food	15, 16	Food, Beverages and Tobacco
Textiles	17, 18	Textiles and Textile Products
Leather	19	Leather, Leather and Footwear
Wood	20	Wood and Products of Wood and Cork
Paper	21, 22	Pulp, Paper, Paper , Printing and Publishing
Fuel	23	Coke, Refined Petroleum and Nuclear Fuel
Chemicals	24	Chemicals and Chemical Products
Plastics	25	Rubber and Plastics
Non-metal	26	Other Non-Metallic Mineral
Metal	27, 28	Basic Metals and Fabricated Metal
Machinery	29	Machinery, n.e.c.
Electrical	30-33	Electrical and Optical Equipment
Vehicles	34, 35	Transport Equipment
Manuf	36, 37	Manufacturing, n.e.c.; Recycling
Utilities	E	Electricity, Gas and Water Supply
Constr	F	Construction
VehicleSale	50	Sale, Maintenance and Repair of Motor Vehicles and Motorcycles; Retail Sale of Fuel
WholeSale	51	Wholesale Trade and Commission Trade, Except of Motor Vehicles and Motorcycles
Retail	52	Retail Trade, Except of Motor Vehicles and Motorcycles; Repair of Household Goods
Hospitality	H	Hotels and Restaurants
InlTrans	60	Inland Transport
WaterTrans	61	Water Transport
AirTrans	62	Air Transport
OtherTrans	63	Other Supporting and Auxiliary Transport Activities; Activities of Travel Agencies
PostTele	64	Post and Telecommunications
Finance	J	Financial Intermediation
RealEstate	70	Real Estate Activities
Business	71-74	Renting of M&Eq and Other Business Activities
PublAdm	L	Public Admin and Defence; Compulsory Social Security
Education	M	Education
HealthSoc	N	Health and Social Work
OthServ	O	Other Community, Social and Personal Services

A.3 Data coverage in WIOD13

Examining the national Input-Output tables in WIOD (2013 release) for all countries and years, the following 7 country-sectors have zero reported inputs for all years in the 1995-2011 period. This corresponds to 0.5% of all sectorial time-series in WIOD13, with 30 out of 34 sectors (88%) having complete data for all countries and years. Although these definitely constitute missing data, these were nevertheless treated as zero values when determining the pair-wise dissimilarity between two fingerprints.

Country	Sector (ISIC3)
China	VehicleSale (50)
Cyprus	Fuel (23)
Indonesia	VehicleSale (50)
India	PublAdm (L)
Luxembourg	Leather (19)
Luxembourg	Fuel (23)
Malta	Fuel (23)

Contrasting these 7 sectorial gaps in WIOD13, the WIOD 2016 release has a total of 169 sectorial time-series with zero reported inputs for all years in the 2000-2014 period. This corresponds to 7% of all sectorial time-series in WIOD16, with only 28 of the 56 sectors (i.e. half) having complete data for all countries and years. This relatively higher data coverage in WIOD13 than in WIOD16 motivates the former is used throughout this study.

Of the 680 potential fingerprints in WIOD13, i.e. covering 40 countries for 17 years, 27 of these were however excluded from all analyses due to their eigenvalue diagnostics. See Appendix D.1.

Appendix B: Cluster memberships

See project website - www.demesta.com/fingerprinting - to visualize and download the detailed average fingerprints (sectorial up- and downstream means and standard deviations) of respective subset. For the 21-cluster partition using average-link (UPGMA), see the project website.

B.1 Average-link (UPMGA) 5-cluster partitions

A. Business, Construction & Vehicles

Australia 1995-2011, Austria 1995-2005, 2009-2011, Belgium 1995-2011, Bulgaria 2007-2011, Canada 1995-2011, China 1995-1999, Cyprus 1995-2011, Czech Rep. 1995-2004, Germany 1995-2005, 2009-2010, Denmark 1995-2011, Spain 1995-2011, Estonia 2007-2011, France 1995-2011, Great Britain 1995-2011, Greece 2002-2011, India 2004-2011, Italy 1995-2011, Japan 1995-2011, Latvia 1998-2011, The Netherlands 1995-2011, Poland 2008-2011, Portugal 1997-2008, Russia 2003-2011, Slovakia 1995-2003, 2006-2008, Slovenia 1995-2011, Sweden 1995-2011, USA 1995-2011

B. Metal & Electrical

China 2000-2001, 2003-2011, Czech Rep. 2006-2008, 2010-2011, Estonia 1998-2006, Finland 1995-2011, Hungary 1997-2011, Ireland 1995-2011, South Korea 1995-1998, 2000, 2004-2011, Mexico 1995-2011, Malta 1995-2011, Taiwan 1995-2011

C. Agriculture & Food

Bulgaria 1995-2006, Brazil 1995-2011, Estonia 1995-1997, Greece 1995-2001, Hungary 1995-1996, Indonesia 1998-2011, India 1995-2003, Lithuania 1995-2011, Latvia 1995-1996, Poland 1995-2007, Romania 1995-2011, Russia 1995-2002, Turkey 1995-1999

D. Finance (Luxembourg)

Luxembourg 1995-2011

E. Textiles

Indonesia 1995-1997, Turkey 2001-2011

B.2 Average-link (UPMGA) 11-cluster partitions

A1. Business
Australia 1995-2002, Austria 1995-2005, Belgium 1995-2011, Cyprus 1995-2011, Germany 1995-2005, 2009-2010, Denmark 1995-2011, Estonia 2007-2011, France 1995-2011, Great Britain 1997-2011, Greece 2002-2011, Italy 1995-2011, Latvia 1998-2001, The Netherlands 1995-2011, Sweden 1995-2011, USA 1995-2011

A2. Construction
Australia 2003-2011, Bulgaria 2007-2011, Czech Rep. 1997-2004, Spain 1995-2011, Great Britain 1995-1996, Latvia 2002-2011, Poland 2008-2011, Portugal 1997-2008, Slovenia 1995-2011

A3. Vehicles
Canada 1995-2011, Japan 1995-2011, Slovakia 2006-2008

A4. Metal & Fuel
China 1995-1999, Czech Rep. 1995-1996, India 2004-2011, Russia 2003-2011, Slovakia 1995-2000

A5. Utilities
Austria 2009-2011, Slovakia 2001-2003

B1. Electrical (disembedded)
Czech Rep. 2006-2008, 2010-2011, Estonia 1998-2000, 2002-2003, 2005-2006, Finland 1995-2011, Hungary 1997-2011, Ireland 1995-2007, Mexico 1995-2011, Malta 2006-2011

B2. Electrical & Metal
China 2000-2001, 2003-2011, South Korea 1995-1998, 2000, 2004-2011, Taiwan 1995-2011

B3. Electrical (embedded)
Estonia 2001, 2004, Ireland 2009-2011, Malta 1995-2005

C. Agriculture & Food
Bulgaria 1995-2006, Brazil 1995-2011, Estonia 1995-1997, Greece 1995-2001, Hungary 1995-1996, Indonesia 1998-2011, India 1995-2003, Lithuania 1995-2011, Latvia 1995-1996, Poland 1995-2007, Romania 1995-2011, Russia 1995-2002, Turkey 1995-1999

D. Finance (Luxembourg)
Luxembourg 1995-2011

E. Textiles
Indonesia 1995-1997, Turkey 2001-2011

B.3 Average cluster distances

Total number of national fingerprints for each cluster given by N_{FP}; number of countries represented in each cluster given by N_C. See Appendix B1 for cluster membership tables.

Table 1: Average within- and between-cluster distance for the 5-cluster partition derived from the (unweighted) average-link (UPGMA) hierarchical clustering

Cluster name [N_{FP}; N_C]	A	B	C	D	E
A. Business, Construction & Vehicles [359;27]	1.17				
B. Metal & Electrical [137;10]	1.54	1.13			
C. Agriculture & Food [126;13]	1.56	1.69	0.83		
D. Finance (Luxembourg) [17;1]	1.71	1.85	1.88	0.06	
E. Textiles [14;2]	1.87	1.89	1.79	1.93	0.29

Table 2: Average within- and between-cluster distance for the 11-cluster partition derived from the (unweighted) average-link hierarchical clustering

Cluster name [N_{FP}; N_C]	A1	A2	A3	A4	A5	B1	B2	B3	C	D	E
A1. Business [202;15]	0.85										
A2. Construction [84;9]	1.29	0.69									
A3. Vehicles [37;3]	1.40	1.54	0.82								
A4. Metal & Fuel [30;5]	1.43	1.36	1.43	0.87							
A5. Utilities [6;2]	1.72	1.71	1.71	1.38	0.39						
B1. Electrical (disembedded) [80;7]	1.40	1.48	1.50	1.49	1.76	0.96					
B2. Electrical & Metal [41;3]	1.69	1.65	1.49	1.37	1.81	1.29	0.67				
B3. Electrical (embedded) [16;3]	1.80	1.84	1.85	1.80	1.89	1.37	1.43	0.34			
C. Agriculture & Food [126;13]	1.54	1.62	1.69	1.41	1.76	1.59	1.79	1.91	0.83		
D. Finance (Luxembourg) [17;1]	1.61	1.85	1.80	1.89	1.96	1.81	1.90	1.94	1.88	0.06	
E. Textiles [14;2]	1.86	1.89	1.88	1.80	1.92	1.86	1.91	1.96	1.79	1.93	0.29

Appendix D: Additional analyses

D.1 Dominant eigenvalues of WIOD13 fingerprints

A fingerprint for a specific national economy at a specific year consists of two parts, capturing the sectorial prominence of up- and downstream flows, respectively. As specified in the manuscript (see Eq. 1 and 2), upstream prominence is operationalized as the left-hand dominant eigenvector of the added Z and M matrices (labeled as the T matrix), and downstream prominence is operationalized as the right-hand dominant eigenvector of the Z matrix alone. What makes these vectors 'dominant' is that their corresponding eigenvalues are the largest (i.e. λ_{max} in Eq. 1-2) among the set of all eigenvalues. As the up- and downstream vectors are extracted from somewhat different matrices (i.e. T and Z), each fingerprint has two sets of ordered lambda values, for up- and downstream prominences, respectively.

Analogous to the calculation of explained variance in factor analysis, the size of the largest eigenvalue relative to the remaining set of eigenvalues could be indicative of how well the dominant eigenvector captures the main features of the matrix. Two diagnostic measures are used to evaluate the size of dominant eigenvalues. The first measure – *1st share* – captures the share of the dominant eigenvalue with respect to the sum of all eigenvalues. The second measure – *1st/2nd ratio* – captures the ratio between the dominant and the second-largest eigenvalue. As a single country-year fingerprint consists of two separate sets of eigenvalues and eigenvectors, for up- and downstream prominence respectively, four eigenvalue diagnostics were calculated for each fingerprint. (Complete tables with eigenvalue diagnostics for all 680 WIOD13-derived fingerpritns are available on the project website.)

	**1st share**	**1st/2nd ratio**
**Upstream eigenvalues (T)**	$u_{\%}$	u_{1vs2}
**Downstream eigenvalues (Z)**	$d_{\%}$	d_{1vs2}

As the sum of all eigenvalues for a matrix A corresponds to the sum of the diagonal values in A (i.e. the trace of A), the eigenvalues of the various Input-Output matrices (Z and T) depend on the relative sizes of the different economies. To allow for a more direct comparison of the dominant eigenvalues for the upstream (i.e. based on Z) and downstream (i.e. based on T) components, these matrices were first normalized so that the sums of their diagonals were fixed at the size of the matrices (i.e. 34):

$$A_{tracenorm} = 34 \cdot \frac{A}{tr(A)}$$

(where A is either the matrix Z or T, tr(A) is the sum of the diagonal of A, 34 is the size of matrix A, and $A_{tracenorm}$ is the trace-normalized version of matrix A where the sum of the diagonal equals 34)

The extracted left- and right-hand eigenvectors of such a trace-normalized version of the Z and T matrices are identical to those of their non-normalized versions, but the corresponding set of eigenvalues now sums up to the number of sectors in the WIOD13 dataset. This allows for a more direct comparison of the relative magnitudes of 1st and 2nd eigenvalues for the up- and downstream vectors for each country and year.

Using these trace-normalized matrices, the statistics for the first (dominant) eigenvalues for the up- and downstream vectors in each fingerprint for the set of 40 countries and 17 years in the WIOD13 dataset is given in Table 3 below, with the frequency distribution of the first eigenvalues given in

Figure 1 below. Percentages in brackets indicate the 1st share eigenvalue diagnostics: on average, $u_{\%}$ and $d_{\%}$ are 27 and 29 percent respectively, with their medians being slightly lower.

Table 3: Statistics on sizes and shares of dominant eigenvalues for up- and downstream eigenvectors

	**Upstream (using T)**	**Downstream (using Z)**
**Mean**	9.09 (27%)	10.00 (29%)
**Median**	8.15 (24%)	9.17 (27%)
**Min-max**	5.27 – 29.69 (16 – 87%)	6.22 – 30.41 (18 – 89%)

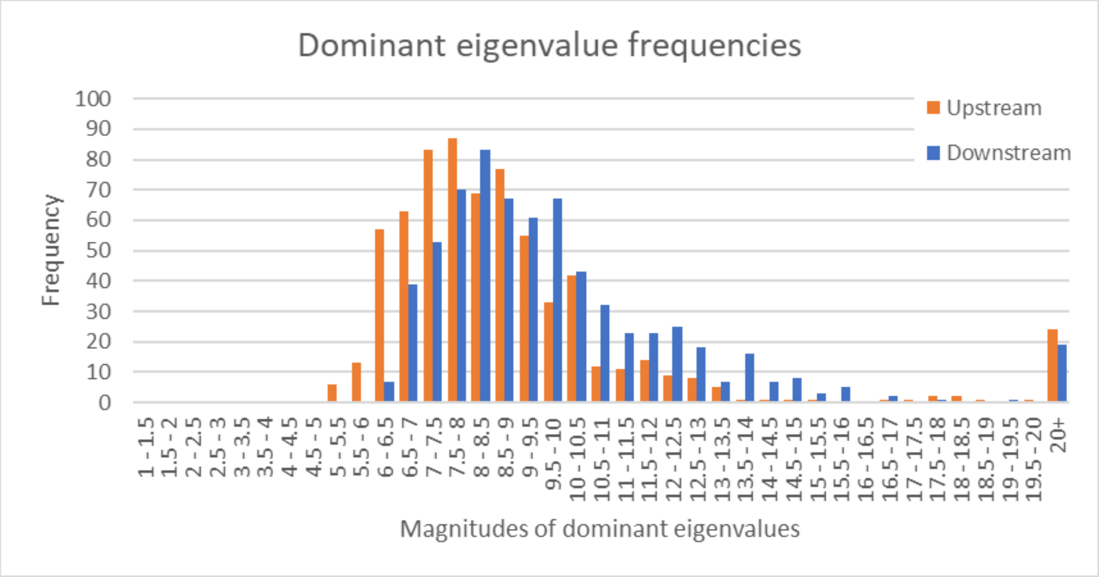

Figure 1: Distribution of dominant eigenvalues for up- and downstream eigenvectors

By dividing the dominant eigenvalues with the second-largest eigenvalues for each of the up- and downstream eigenvectors, we obtain the second eigenvalue diagnostics for up- and downstream eigenvectors: u_{1vs2} and d_{1vs2}. Mean, median and value ranges for these diagnostics are given in Table 4, with their distributions given in Figure 2.

Table 4: Statistics on ratio between dominant and second-largest eigenvalues for up- and downstream eigenvectors

	**Upstream (using T)**	**Downstream (using Z)**
**Mean**	2.05	2.04
**Median**	1.54	1.66
**Min-max**	1.08 – 18.81	1.11 – 19.24

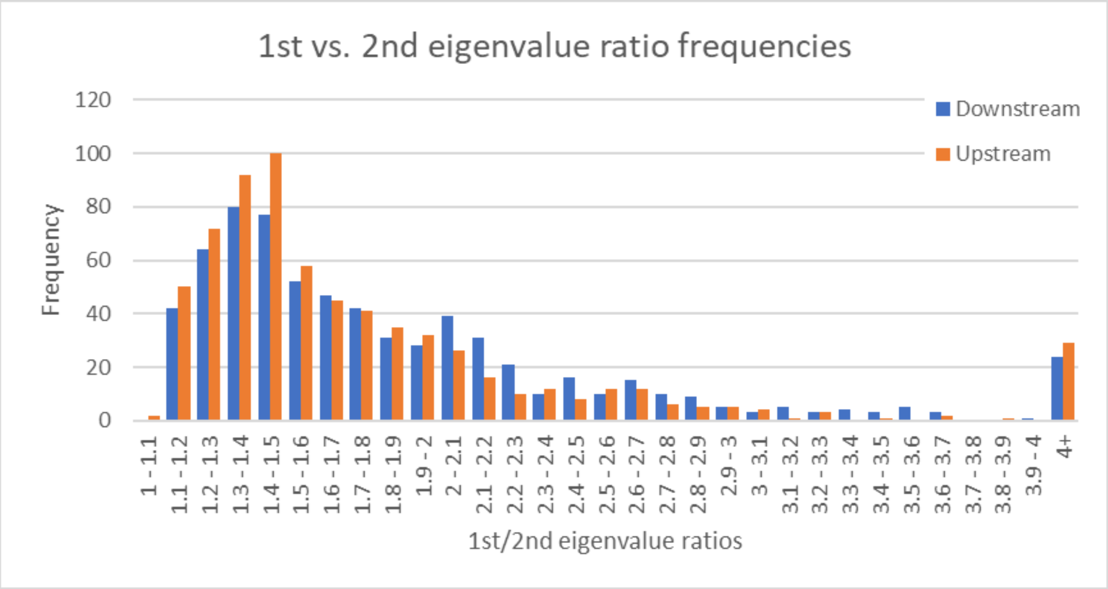

Figure 2: Distribution of 1st vs. 2nd eigenvalue ratios for up- and downstream eigenvectors

The mean and median eigenvalue diagnostics are relatively good, but there are indeed several fingerprints where either one or both of its eigenvectors have corresponding eigenvalue diagnostics that are low. For those where both of the dominant eigenvectors have low diagnostics, Portugal's 1995 fingerprint has the objectively worst diagnostics ($u_{\%}$ = 15.5%; u_{1vs2} = 1.13; $d_{\%}$ = 18.5; d_{1vs2} = 1.20), with Turkey 2000 as a good contender for the second worst ($u_{\%}$ = 17.9%; u_{1vs2} = 1.21; $d_{\%}$ = 18.3; d_{1vs2} = 1.17).

Portugal 1995 and Turkey 2000 stick out with both their diagnostic measures being very low, which is not the case for the vast majority of fingerprints. If we set an acceptable threshold for the 1st share diagnostic measures (i.e. $u_{\%}$ and $d_{\%}$) to 20 percent and a corresponding threshold for the 1st vs. 2nd ratio diagnostic measure(i.e. u_{1vs2} and d_{1vs2}) to 1.25, the number of fingerprints with combinations of acceptable diagnostic measures for their up- and downstream components are as given in Table 5 below.

Table 5: Frequency table for acceptable eigenvalue diagnostic measures

	**$\lvert u_{1vs2} > 1.25\rvert$**	**$\lvert u_{1vs2} < 1.25\rvert$**	$\sum$
**$\lvert u_{\%} > 0.20\rvert$**	502 (74%)	67 (10%)	569 (84%)
**$\lvert u_{\%} < 0.20\rvert$**	90 (13%)	21 (3%)	111 (16%)
$\sum$	592 (87%)	88 (13%)	

	**$\lvert d_{1vs2} > 1.25\rvert$**	**$\lvert d_{1vs2} < 1.25\rvert$**	$\sum$
**$\lvert d_{\%} > 0.20\rvert$**	588 (86%)	64 (9%)	652 (96%)
**$\lvert d_{\%} < 0.20\rvert$**	20 (3%)	8 (1%)	28 (4%)
$\sum$	608 (89%)	72 (11%)	

With Portugal 1995 and Turkey 2000 being part of both the 21 fingerprints with sub-par upstream diagnostics and the 8 with sub-par downstream diagnostics, there are a total of 27 fingerprints with potentially problematic eigenvector components – see Table 6 below. These fingerprints should thus be interpreted with particular care, and they are therefore removed from the analyses presented in the paper.

Table 6: Country-year fingerprints excluded from the analyses

Austria 2006-2008	South Korea 1999, 2001-2003
China 2002	Latvia 1997
Czech Rep. 2005, 2009	Portugal 1995-1996, 2009-2011
Germany 2006-2008, 2011	Slovakia 2004-2005, 2009-2011
Ireland 2008	Turkey 2000

These thresholds could be criticized as being too liberal, with the implication that one should be even more careful in interpreting the dominant eigenvectors and the obtained fingerprints of up- and downstream sectorial prominence for a larger set of fingerprints. Still, even with sporadically low eigenvalue diagnostic measures for particular fingerprints, these do not seem to interfere much with results from comparative fingerprinting analysis. The case in point is the cluster analysis done in this study (see Section 3.1 in manuscript, particularly Figure 11): although specific fingerprints indeed have rather poor eigenvalue diagnostics, these fingerprints nevertheless seem to capture enough structural features of their economies to result in longitudinally consistent clusters that make sense.

D.2 Analyzing structural trajectories using multidimensional scaling: the case of the European Union 1995-2011

Given a set of fingerprints and their pairwise dissimilarity/distance metrics, the first case study in the article demonstrates how such a matrix could be analyzed using hierarchical clustering. Whereas this approach provides a way to separate entities into discrete, nominal subsets, such distance data could also be approached using the family of techniques for dimensionality reduction, which maps dissimilarity/distance data into (typically) two or three dimensions. Classical multidimensional scaling (MDS; also known as principal *coordinates* analysis) constitutes one such alternative tool for mapping out similarity patterns and potential latent variables, as well as tracking the developmental trajectories of such structures over time, in a more continuous, non-categorical way.

Building on the second case study in the article, concerning the Eastern enlargement of the European Union, all ten Central- and East-European countries were initially included (i.e. Bulgaria, Czech Republic, Estonia, Hungary, Latvia, Lithuania, Poland, Romania, Slovakia, and Slovenia), together with five of the six founding members of the European Union (i.e. Belgium, France, Germany, Italy, and the Netherlands). Covering 15 countries over 17 years, though here also excluding 12 country-year fingerprints due to low Eigenvalue diagnostics, Euclidean distances for each pair of these 243 country-year fingerprints were calculated. Classical metric multidimensional scaling (MDS) was applied to this dissimilarity matrix to extract both 2- and 3-dimensional solutions. Evaluating these solutions with the Kruskal stress index, the 2-dimensional solution resulted in a high stress of 0.49, dropping to 0.30 for the 3-dimensional solution. Further exploration revealed that it was particularly Estonia's inclusion that contributed to this stress. Redoing the MDS with Estonia excluded, i.e. with a total of 14 countries and 226 country-year fingerprints, the Kruskal stress went down to 0.43 for the 2-dimensional solution and 0.27 for the 3-dimensional solution. As the 3-dimensional solution lies below the generally acceptable threshold (0.3), this solution was chosen for further exploration.

Calculating 3-year simple-moving-average coordinates for all years (using 2-year averages for 1995 and 2011, respectively), Figure 3 depicts the first two dimensions of the 3-dimensional MDS solution.

Whereas this figure can be perceived as looking at the 3-dimensional solution from the side (like the side of a cube), the supplementary figure (Figure 4) looks at the same data but from "above" (like you are leaning forward into the paper surface and looking down). Whereas the 3rd dimension indeed is necessary to bring down the Kruskal stress index to reasonable levels (i.e. 0.27), the first two dimensions (Figure 3) does seem to allow for some interesting interpretations. First, corroborating findings from the two case studies in the article, the production structures of these five Western economies are relatively similar and stable over time. Their structural trajectories are overall unidirectional, more linear, and seemingly ending up closer to each other in 2011 compared to 1995. In contrast, most of the 'Eastern' production structures experience dramatic structural transformations. The structural trajectories of Bulgaria, Latvia, Hungary and Poland are particularly notable, traversing large distances in this 'structural space'. Lithuania, and perhaps also Hungary, seem to experience retrograde structural trajectories, but Lithuania, together with Romania, do not move very far away from their starting position. This is in line with the sequence index plot of the 11-type classification (see Figure 11 in manuscript): of the seven 'Eastern' countries starting off as 'Agriculture & Food'-type economies in 1995, only Lithuania and Romania remain as this type throughout the 1995-2011 period.

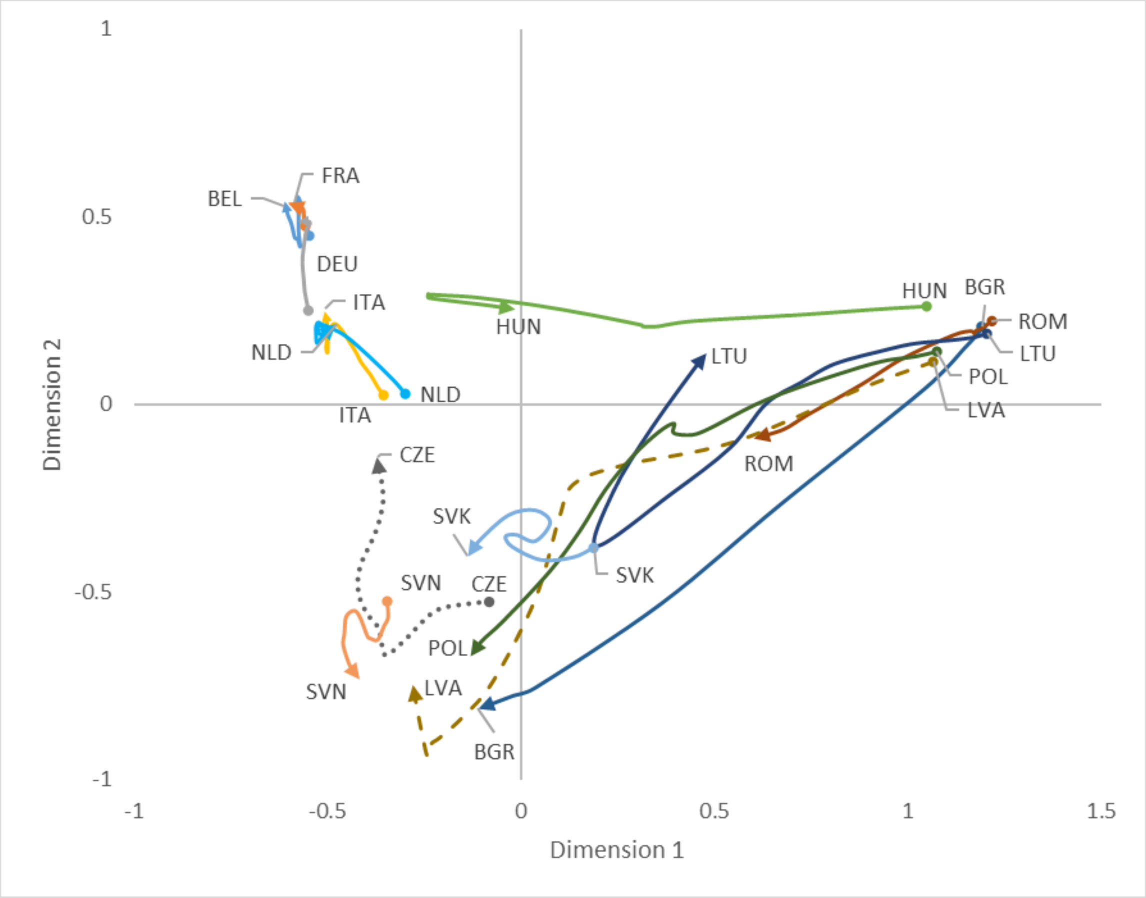

Figure 3: Classical multidimensional scaling (dimension 1 and 2) of structural trajectories in the European Union, 1995-2011

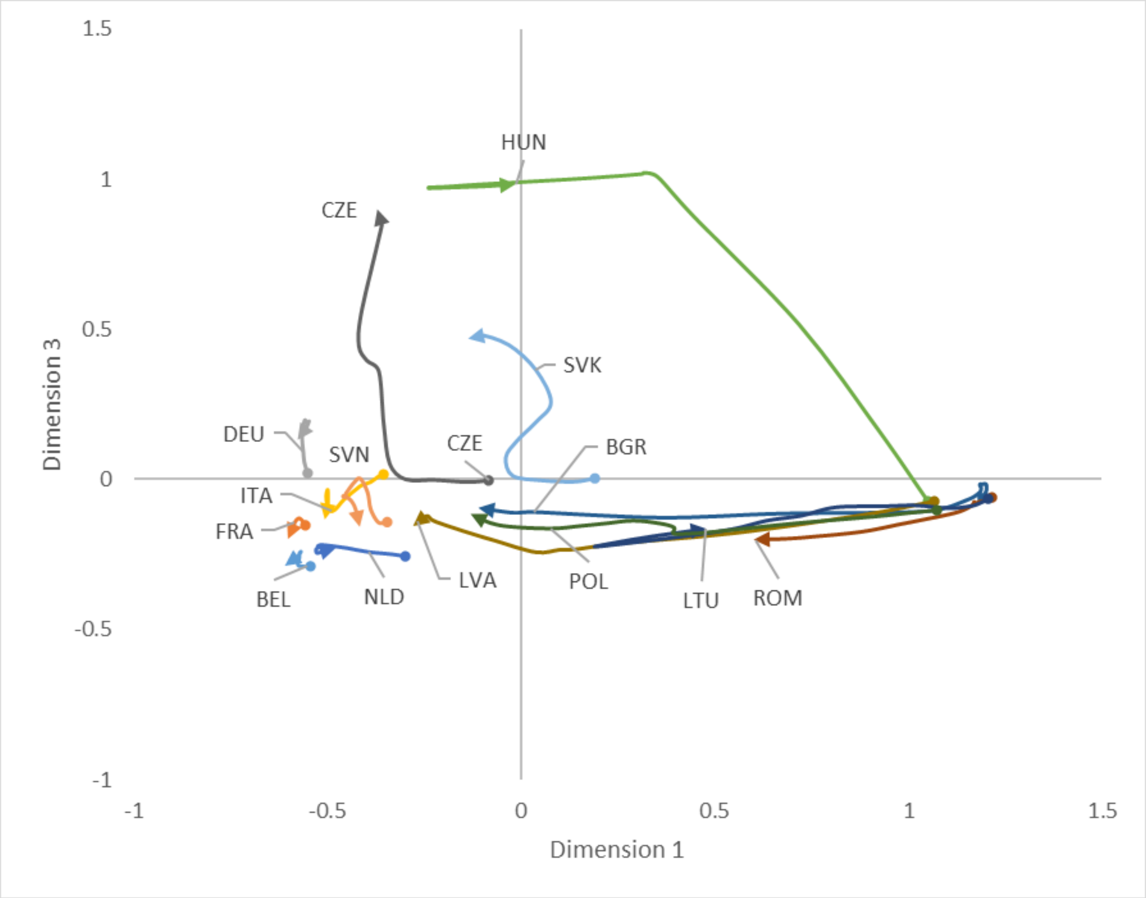

Figure 4: Classical multidimensional scaling (dimension 1 and 3) of structural trajectories in the European Union, 1995-2011

Although both Hungary and Czech Republic exhibit movement towards the 'Western' cluster, a notable aspect of Figure 3 is the lack of East-West structural convergence. As concluded from the longitudinal plots in the paper (see Figures 16 and 17 in the manuscript), the transformations of the 'Eastern' production structures did not markedly affect the average structural dissimilarity between the two regions. This finding is well reflected by the first two dimensions of the MDS solution above: despite a significant transformation of several of the Eastern production structures during this period, they seem to remain equidistant to their 'Western' counterparts. Additionally, albeit relatively short, the trajectories of the 'Western' structures seem to move them further away from their Eastern neighbors.

Multidimensional scaling could potentially be a useful supplement to the existing tools for fingerprint analysis proposed in this article. However, similar to all such techniques, it is of course imperative that relevant goodness-of-fit measures, such as the Kruskal stress index here, remain within acceptable boundaries. In the context of fingerprinting, tentative explorations of the WIOD13-based fingerprints seem to indicate that three dimensions are needed to arrive at such acceptable levels. Thus, the visualization and interpretation of such rescaled data should then preferably instead be done through interactive and/or virtually augmented/VR tools.
