# Supplementary material for: Transformations, trajectories, and similarities of national production structures: A comparative fingerprinting approach
Source: PLoS One. 2023 Dec 19;18(12):e0295568. doi: 10.1371/journal.pone.0295568 (PMC10729992; doi:10.1371/journal.pone.0295568)
Supplement: Attachment — Submitted filename: Response to reviewers.docx [file pone.0295568.s002.docx]

# Author's response to reviewers

I am grateful for the valuable feedback and suggestions from the two anonymous reviewers. In what follows, I have responded to the comments from the two reviewers. As Reviewer 2 provides both major and minor issues, whereas reviewer 1 provides only minor points, my response begins with the major points raised by Reviewer 2, followed by the minor points of Reviewer 2 and 1.

## Reviewer 2

*- Please improve the discussion concerning the degree of novelty of the proposed framework in light of the current literature. You may want to consider also the following articles:*

*Cerina et al. (2015), Liu & Tsyvinski (2020), Wang et al. (2020), Giammetti et al. (2020).*

Thank you for your suggestion and these references: I have read Cerina et al (2015) and Liu and Tsyvinski (2020) before, but was not aware of Wang et al (2020) and Giammetti et al (2020), which I have now also digested. Thank you: I am always interested in finding potential papers that propose novel approaches for spatio-temporal comparisons of national production structures.

These four papers do in various ways indeed connect to Dietzenbacher’s approach of using left- and right-hand eigenvectors to analyze production structures as expressed in input-Output data. In Liu and Tsyvinski’s paper, the analysis explores the IO-derived tech coefficients (A) using left- and right-hand dominant eigenvectors, i.e. similar to what Dietzenbacher does. Cerina expands this to a multi-regional setting, applying left-hand eigenvector to capture backward linkages in a multi-national setup of input-output flows. Wang et al’s study (which I wasn’t aware of) similarly looks at tech coefficients, applying left- and righthand dominant eigenvector to examine sectorial rank orders for an individual country (China). Giammetti et al similarly looks at an individual country – UK – here looking at the actual flow matrices (Z) and applying the Dietzenbacher approach as a comparison with the PageRank centrality approach Giammetti uses.

Still, despite these ties to the Dietzenbacher article (and subsequent reiterations), I argue that these studies differ from the aim and purpose of the fingerprinting heuristic. Cerina et al’s paper views the global network of interconnected national industries as a whole as the unit of analysis. Despite obvious increasing international integration, their community detection approach confirms that national systems of production (still) seem to constitute relevant units of analysis and comparison. Cerina et al’s comparative analysis of individual country-industries is interesting – like a Dietzenbacher for the whole GMRIO network, where they identify top 20 industries in the global production network as a whole. This is however not a comparative analysis of national production structures as individual wholes. Many papers indeed look at the embeddedness of individual country-industries, and clusters thereof, like this, but this paper doesn’t provide a way to compare the production structures of countries. The only potential comparison is whether their respective industries appear on similar ranking, but that would then also be size-dependent. So even if this indeed involves a back-and-forward-linkage Dietzenbacher-style of analysis, which is interesting per se, it is not aimed at comparing the production structures of countries as individual units of analysis.

In a similar vein, Liu and Tsyvinski’s study applies Dietzenbacher’s approach of left- and right-hand dominant EV’s, doing this for an individual economy’s technology coefficients. This is an interesting study, but equally does not provide a heuristic for the comparative study of national production structures. Wang et al similarly look at the tech coefficients of an individual economy, i.e. no country-wise comparison in the same vein as the fingerprinting approach. And that is of course not the purpose of Wang et al.’s paper: similar to the other papers I have found (including these four), the analysis is concerned with issues such as the rank orders of individual industrial sectors, for an individual country (as here w.r.t. to co2 emissions) or for all sectors in a multiregional production system (like Cerina et al).

Still, these papers are indeed relevant to mention and referencing in the manuscript. Cerina et al and Giammetti et al have been added to the network-analytical-approach section of the literature review (line 206), and Liu and Tsyvinski and Wang et al is referenced when introducing the Dietzenbacher left/right approach, as these are more novel iterations of this approach. However, based on the reasoning above on what these papers do, I do argue that neither of these papers (nor any other paper that I have found) propose novel ways for the comparative analysis of the structural features of production structures of individual countries in a similar vein as what the fingerprinting heuristic aims to do.

*- Up-stream and down-stream “finger prints” seem to be fairly correlated to one-another. One may wonder whether the results may be severely altered if just one of the two indices is considered in computing the Euclidean distance (Eq. 3). Similarly, the author may want to test other ways to compare two vectors. For instance, the author might consider using cosine similarity emphasizing similarities and differences w.r.t. the Euclidean distance already used.*

These are great points, both of which I have explored during the development of the heuristic. By correlating the up- and downstream parts of each fingerprint, I get a mean correlation of 0.56, ranging from -0.132 to 0.998, and with [Q3-Q1] = [0.83 – 0.35]. (Not surprisingly, Luxembourg tops the list in correlations between up- and downstream profiles!). If I were to just look at either of these parts, i.e. either the up- OR the downstream part, and determine pairwise dissimilarities based on that alone, the obtained results are different than compared both with the combined (default) approach and when comparing up- vs. downstream parts.

The electrical-sector oriented countries exemplify these differences well. All these countries share prominent upstream electrical sectors: i.e. lots of upstream (input) to this sector in Malta, China, S Korea, Taiwan, Finland, Ireland, Mexico, Hungary. If I were to determine dissimilarities and clusters based on upstream profiles only, all these countries would form a joint and seemingly similar structural subset. However, as evident in the 11- and 21-cluster partitions in Figure 8, their downstream profiles are quite different. Such distinctions wouldn’t emerge if the dissimilarity and clustering analyses were done using downstream profiles only: then only a handful of country-years with domestically embedded electrical sectors (Malta, parts of Estonia, Ireland 2009-11) would emerge as electrical-sector economies.

Thus, we need both up- and downstream sectorial properties to be able to capture such distinctions and variations among electrical-sector-dominated economies, e.g. that Mexico and Hungary indeed are quite similar to each other, and that only a handful of electrical-dominated economies managed to really embed these footloose industries into their domestic economies and other sectors at large. Albeit the average correlation might be somewhat high, both the up- and downstream vectors are necessary to make crucial distinctions between countries, such as how various sectors are domestically embedded or not.

That said: this doesn’t rule out that certain research designs might be focused on up- or downstream profiles only: if fitting for a particular research question, it could make sense to analyze these individually. E.g. if one is solely looking at electrical-oriented economies, wanting to compare their domestic embeddedness (where we know that they all have large upstream flows for that sector), then it might make sense to determine dissimilarities on the basis of domestic downstream flows only. But that must then be left for subsequent studies and proposed modifications to the herein proposed approach.

Regarding alternative metrics for comparing fingerprints: Euclidean distances indeed only constitute one type of comparative measure – cosine dissimilarity and Pearson correlations are two other viable alternative metrics, both of which I have previously experimented with. However, the choice of Euclidean distances over cosine similarity was based on the nature of the data that is to be compared. The up/downstream prominence measures, i.e. the directed dominant eigenvectors, are unit measures, where the actual absolute values do signify sectorial prominence. E.g. if the upstream prominence of a sector for country A is 0.5 and the corresponding measure for country B is 0.25, this should be interpreted that the prominence of this sector for country A is larger than the corresponding sectorial prominence for country B (which surely is linked to the prominence and interconnectedness of all sectors). Yet, for the interpretation of individual fingerprints, the magnitude of these prominence values should reasonably be comparable across countries and years. The cosine similarity captures the angle between N-dimensional vectors, while ignoring the actual magnitudes (lengths) of these vectors. Thus, if two countries have identical fingerprints but where their prominence values differ by a scaling factor, the cosine similarity would indicate that these are identical. For instance, that the up- and downstream prominence of Luxembourg’s financial sector is so dominant and large should indeed matter. If this is compared with a hypothetical country whose financial sector prominence is only a tenth that of Luxembourg, also having overall low prominence of all other sectors, the cosine similarity measure would categorize Luxembourg and this country as likely identical (as their prominence vectors would point in the same direction, though with vastly different lengths). (Correlation metric would equally ignore the actual magnitudes of sectorial prominences, only looking at the shape of the sectorial distribution rather than actual prominence measures).

Contary to cosine similarity (and correlations), the Euclidean distance measure captures such differences in sectorial prominence. Expressed differently: cosine similarity ignores the magnitudes/lengths of vectors and only looks as the angle between them. If there was a vector with a large magnitude in the same direction as as a vector with a short magnitude, they would have a cosine similarity of 1, even though the Euclidean distance would be quite large.

That said, although Euclidean distances is the measure I propose in this article, I am not arguing that the Euclidean distance metric is the most ideal measure in all circumstances. When I worked on this project a few years ago, I did also test correlations and cosine similarity measures, and I recall I got quite similar clustering results back then. However, the Euclidean distance metric came across as the most intuitive, especially as distances then can be conceptualized as distances between two points (economies) in a hypercube. It also works well to visualize, e.g. as in Figure 4, where the Euclidean distance between two economies can almost be seen as the colored bars in this figure.

*- Regarding the second case study, the author may also be willing to consider a deeper discussion of his findings in light of the economic theory and the recent history of the Eastern European countries, which appears to be missing. Moreover, the analysis performed by the author may benefit from a ranking of Eastern European countries according to their degree of convergence to the Western countries' fingerprints.*

This is good feedback and something I initially had planned for the article, but I simply ran out of paper real estate to fulfill this particular avenue (as well as other theoretical extensions I had in mind). The original idea for this article came during my participation in a larger EU project on economic development in Eastern Europe, i.e. where I was explicitly looking at different ways to conceptualize and formally operationalize what we mean with structural transitions, convergence and divergence in the context of EU accession, that made theoretical sense. I do pay respect to both the Balassa tradition of economic integration (which was mainstay theory in that original FP7 project) as well as the market integration ideas by Krugman and Venables, and it would particularly be nice to have a deeper introduction to Krugman’s 1991 idea that for overall welfare, “close integration is good, but a limited move toward integration may hurt” (Krugman 1991:89): there is a lot of potential discussion about the fingerprinting findings on structural convergence with the west. It would also be really interesting to go a few steps deeper: to compare the structural dissimilarities, both on the aggregate East-West regional level as well as for individual countries between East and West, and also within the Eastern subset, with other national attributes such as value-added, export compositions, GDP growth etc – not to mention national policy differences. However, due to lack of paper real estate, I feel constrained to dig into all this literature and existing arguments (which is a huge corpus with many different strands), but rather merely acknowledge and refer to these original theoretical entry-points, i.e. Balassa and Krugman/Venables, which I do in the opening sentence of the second case study.

However, as a deeper discussion on EU integration and Eastern expansion might be too comprehensive (and certainly run the risk of being quite selective) for this manuscript, I have added a part at the end of case study 2, where I claim that the findings from the fingerprinting approach – i.e. transformation without convergence - could have implications for existing theories of EU integration, with references to some papers that represent a couple of such theoretical frameworks. With this, I do hope that future studies of a more theoretical and regional nature will dig into these findings, linking them more thoroughly to these theoretical frameworks.

*- Captions should be improved as it needs to be self-contained. For what concerns Figure 1, please provide a short description of the different nodes.*

Excellent, thank you. I have checked the detail levels of captions in existing PloS ONE articles and updated the captions in my manuscript, following the PLOS ONE style guidelines that I received. Each caption now has a main text (in bold) and a more detailed description, to make them all self-contained.

*- Different font in “Dietzenbacher’s” , l. 240*

Checking line 240 (or line 244), I don’t see that the font is different here: Calibri (Body) all across. If this is something I have missed, I am certain this will be fixed at the would-be publishing stage.

*- Please clarify in the text the use of the symbol ⊕ as it might be ambiguous. 292.*

Thanks: mentioned what this symbol means in this context.

*- Please clarify according to which criterion “…the mean and median diagnostics are satisfactory”, l.356.*

This is a good point: my chosen thresholds for what are acceptable values for these two diagnostics are admittedly somewhat arbitrary chosen. On average, having a first eigenvalue that is twice the size of the second eigenvalue could indeed indicate that the first eigenvector should constitute an adequate representation of the structural features. Yet, what is noticeable in the analyses that follow is that the first eigenvectors indeed still seem to be “stable” throughout: despite intermittently low EV diagnostics for particular country-years, there is an evident consistency with respect to cluster membership. The sequence index plot (Figure 10) supports this: those country-year excluded (I.e. with relatively low EV diagnostics) are mostly occurring where countries transition between structural types. Yet, if I also include these country-years, these would then either remain in their surrounding types (Germany, Czech rep, China and S. Korea) or connect to either the preceding or subsequent type (e.g SVK, AUT, LVA, TUR). As such, setting the specific EV diagnostics thresholds differently do not change the overall clustering results.

However, in response to the reviewer’s comment, I have refrained from using the term ‘satisfactory’ in this part, instead reformulating this part by noting that yes, on average, the 1^st^ eigenvalue is more than twice that of the 2^nd^ eigenvalue, but that there are several fingerprints where the diagnostics could be better. Although it can indeed be debated what a suitable EV diagnostics threshold should be, the point I make here (and in Appendix D.1) is that the results evidently are stable despite this. (Yet, it feels prudent to indeed include the EV diagnostics sector in the paper, and actually remove a couple of fingerprints, even though the impact on clustering results seems marginal).

## Reviewer 1

*- What is the difference between Dissimilarity index and the known measure of Structural Equivalence (measured by Euclidean Distances See Burt 1988, Burt and Carlton, 1989, who also analyzed Input-Output tables):*

*Burt, R. S. (1988).* *‘‘The Stability of the American Markets,’’ American Journal of Sociology 94, 356–95.*

*Burt, R. S. and Carlton, D. (1989). ‘‘Another look at the network boundaries of American markets,’’American Journal of Sociology 95, 723–753.*

Given a network, the notion of structural equivalence (as with blockmodeling in general and other types of equivalences) is concerned with identifying nodes that fulfill the same structural role in a network (see, e.g., Nordlund 2016; 2020; Nordlund and Ziberna 2019). For two nodes to be structurally equivalent, they have similar patterns of ties to alters, i.e. both with respect to existing as well as missing ties. In the study of Burt (as well as many early papers building on White et al’s foundational ideas), so-called indirect measures of structural equivalence were first calculated, e.g. by comparing the row- and column vectors of two nodes in a sociomatrix. When using Euclidean distance to compare these vectors (as Burt does in these two papers), the indirect measures of structural equivalences are measures of dissimilarity. At zero dissimilarity, two nodes are perfectly structurally equivalent, where increasing values indicate less structural equivalence. These measures can then be analyzed, e.g. using FA/PCA (as Burt is doing with his MDS) or through agglomerative hierarchical clustering, to identify subsets of nodes that are more structurally equivalent to each other. In the context of doing this on national IO data, i.e. as Burt does in these papers, it is possible to group sectors within a national economy on the basis of their structural similarities, i.e. on the basis that they share similar tie patterns to other sectors. One can also use these dissimilarity matrices to do a multidimensional scaling (such as in Burt and Carlton, 1989, . 728, Figure 1), where extracted dimension potentially could tell a story about roles in the networks.

Whereas the structural equivalence dissimilarity values in Burt’s studies indicate how structurally equivalent two economic sectors in a singular economy are with each other, i.e. to what degree sectors have similar patterns of ties to all other sectors in the individual economy, the pairwise dissimilarity measures in the fingerprint heuristics tell something about the degree of structural similarity between a set of multiple countries (and years). Thus, the pairwise dissimilarity measures for the indirect measures of structural equivalence that Burt et al uses (see equation in Burt and Carlton, 1989, p. 724), reflects how similar the relational patterns to alters of two sectors are within an individual economy. This is in contrast with the pairwise dissimilarity measures of the fingerprinting heuristic, which reflects how similar two fingerprints (each representing the complete networks of national production for a particular country and year) are. Both of these dissimilarity matrices can be analyzed in the same way, e.g. either by agglomerative hierarchical clustering (which I do in Case study 1) or by multidimensional scaling, which Burt does in these two papers (and which I also do in the study I placed in the Appendix). But they indeed represent two different aspects.

In one of the previous research designs when I developed the fingerprinting heuristic, one of the ideas I tested was to do structural equivalence blockmodeling of each of the country-year Input-Output tables. Contrary to Burt’s approach, I then used correlations rather than Euclidean distances as an indirect measure of structural equivalence. From this, I then did agglomerative hierarchical clustering to prune out another type of structural fingerprint, i.e. to map out the underlying block image (which can be seen as a latent functional anatomy of a network; see Nordlund 2020 (SocNetJ)) and then compare these fundamental structures with each other and create a taxonomy of structures based on that. However, similar to the findings of Dietzenbacher and subsequent scholars, the blockmodeling approach I tested tended to capture quite similar structures for all tested economies. This is not surprising: it is unlikely that the automobile and electrical sectors have a lot of inputs from the agricultural sector, and it is quite expected that raw material sectors are structurally equivalent due to their particular roles in virtually all economies. Thus, results were a bit like the IO triangulation papers on sectorial hierarchies: it is feasible to expect certain fundamental structures to exist in a modern economy.

*- Why not produce a matrix of Similarity Index between all or most of the data points? Is it possible then to extend the method to include more than merely pairwise comparison? (The matrix can be compacted into simple map by data reduction techniques)*

This is indeed what I produce in Case study 1: the input to the agglomerative hierarchical clustering I do constitutes such a matrix of dissimilarity measures (i.e. Euclidean distances), where each measure represents the dissimilarity of two economies (country-years). This matrix is available for download on the project website: <https://demesta.com/fingerprinting/data.php>

The clustering analysis in case study 1 indeed goes beyond merely pairwise comparisons. The pairwise comparison of Sweden and Denmark in 1995 (Figure 4) is indeed a pairwise comparison, but the cluster analyses do involve the whole set of all 212,878 pairwise dissimilarity measures. Regarding visualizing such data using data reduction techniques, such as multidimensional scaling, this is also something I do in the final analysis in Appendix D.2. In this study, I use all dissimilarity measures for the complete set of dissimilarity measures for all 15 included EU countries in case study 2, for all years in the 1995-2011 period, which allows me to track the structural trajectories of countries over time. However, due to the complexity of this data, the Kruskal stress index remains quite high up until the 3-dimensional visualization, but this visualization indeed reinforces my findings on the orbital trajectory of East-European countries after accession: transformation without convergence.

Using the dissimilarity matrix I provide on the website, it is possible to do similar multidimensional analyses using other selection of countries; that should be easy to do following the dissimilarity measures I provide, and the analytical approaches I present in both case studies as well as the auxilliary MDS-based study in appendix D.2.

*- What about the relations between structural dominance and performance?*

*Other works have linked network analytic tools of I/O, derived power position, and dependent variables (mostly profitability). See, for example, Talmud, I., “Relations and Profits: Imperfect Competition and Its Outcome”, Social Science Research, 23, 1994: 109-135. Talmud, I., “Industry Market Power, Industry Political Power, and State Support: The Case of Israeli Industry”, Research in Politics and Society, 4: 35-62, 1992.*

This, as well as the final (4^th^) question of Reviewer #1 are both really interesting, and something I definitely would like to dig deeper into moving forward. Whereas the fingerprint heuristic provides a novel way to compare national production structures, arguably producing interesting results, the subsequent step is to link such observed similarities, trajectories and transformations to external data. How are particular production-structural types linked to GDP growth, unequal exchange, international trade and power asymmetries, national income distributions, value-added capture, and world-system positionality? On a longitudinal basis: to what degree are certain national policies linked to structural transformations of said national economies? Do structural transformation of a national production structure link up with how a national economy is embedded in world-system structures? Do certain fingerprints correspond to the beneficial and detrimental sides of Emmanuelian unequal exchange?

Yet, to actually address such associations in this paper is, I believe, not possible due to article real estate, and the importance of first presenting the heuristic itself (along with two case studies). Although I do have a short discussion about how national transitions between different structural types relate to specific economic-historical events (see page 25), such questions indeed deserve more space. However, for a paper that introduces the technicalities of a novel way to capture and compare national production structures in the transnational production regime, I deem it unfeasible to dig further into substantive aspects: this is the first iteration of this proposed heuristic, and as I express in the conclusion, it feels imperative to first let it be examined and evaluated by the scholarly community, before using it to draw any larger substantive conclusions.

However:

*- 4. What is the primacy of the “fingerprint” approach to external network analyses of the world system?*

This is indeed the question that in a previous draft of this manuscript constituted a 3^rd^ case study. As my own scholarly background is world-system analysis (see, e.g., Nordlund, 2006; 2010; 2014; 2018), I find it very interesting to link together the fingerprinting findings with findings from the world-system network-analytical papers that the reviewer mentions here. Specifically, by providing insight on how domestic production structures relate to world-system positionality (e.g. through blockmodeling analysis of international trade flow networks; see Nordlund 2010; 2014; 2016; 2018), the fingerprint heuristic could potentially complement the picture provided by the more exogenously oriented world-system and dependency analytical approaches. As the fingerprinting heuristic takes transnational production into account, i.e. as originally proposed by the Global Commodity Chain tradition of Wallerstein and Hopkins, I deem that the approach is quite compatible with these more global-structural network analyses of contemporary world-system researchers, such as Mahutga, Talmud, Kick, Lloyd, Smith, and my own humble research contributions to modern world-system analysis. For instance: given a blockmodeling study of the contemporary world-economy (see, e.g., Nordlund 2014), it would be really interesting to see whether identified clusters (i.e. world-system trimodal strata) relate to certain types of fingerprints. It would equally be interesting to see whether transitions between world-system strata, e.g. from semiperiphery to core (as captured by international relation networks, such as trade) is linked to structural transformations within the countries experiencing such transitions.

However, similar to question 3 above, this current manuscript is primarily a methodological piece, where I introduce a novel heuristic for comparing national production structures in a contemporary transnational production regime (i.e. which is the setting of the modern world-system of today). If the approach is deemed viable by the research community working on these topics, my ambition is definitely to start applying this approach to ask more substantive questions, both with respect to extant theories of economic integration in the European Union, questions about structural properties vis-à-vis measures of performance and power, and how the structure and dynamics of national production structures are linked to world-system relations.

*- 5. Table 3 should be moved to the Appendices.*

Great idea: done.
